# Supplementary material for: Electronic Nicotine Delivery Systems Use and Periodontal Health—Findings from the Population Assessment of Tobacco and Health Study
Source: Healthcare (Basel). 2023 Dec 21;12(1):25. doi: 10.3390/healthcare12010025 (PMC10779217; doi:10.3390/healthcare12010025)
Supplement: Supplementary file 1 [file healthcare-12-00025-s001.zip › healthcare-2750571-supplementary.pdf]

Table S1. Covariates and oral health outcomes variables

Variable	Response	
	PATH Study	This Study
**Alcohol use**		
1. Have you ever used alcohol at all, including sips of someone's drink or your own drink?	☐ Yes ☐ No	☐ Yes ☐ No
**Other tobacco Use**		
2. Have you ever smoked any cigar product?	☐ Yes ☐ No	☐ Yes ☐ No
3. Have you ever smoked a pipe?	☐ Yes ☐ No	☐ Yes ☐ No
4. Have you ever smoked a hookah?	☐ Yes ☐ No	☐ Yes ☐ No
5. Have you ever used snus pouches?	☐ Yes ☐ No	☐ Yes ☐ No
6. Have you ever used smokeless tobacco?	☐ Yes ☐ No	☐ Yes ☐ No
7. Have you ever used dissolvable tobacco?	☐ Yes ☐ No	☐ Yes ☐ No
**Behaviors and Attitudes**		
8. Have you ever had your teeth cleaned by a dentist, hygienist, or other health professional?	☐ Yes ☐ No	☐ Yes ☐ No
9. In the last seven days, how many times did you use dental floss or any other device to clean between your teeth?	------------------	☐ ≥ 7 times ☐ < 7 times
10. Based on what you believe, how much do you agree or disagree with the following statement? Smoking can cause mouth cancer in smokers.	☐ Strg. Agree ☐ Agree ☐ Neither agree nor disagree ☐ Disagree ☐ Strg. Disagree	☐ Agree ☐ Neither agree nor disagree ☐ Disagree
**Outcome measures**		
11. Have you ever been told by a dentist, hygienist, or other health professional that you have gum disease?	☐ Yes ☐ No	☐ Yes ☐ No
12. Have you ever been told by a dentist, hygienist, or other health professional that you lost bone around your teeth?	☐ Yes ☐ No	☐ Yes ☐ No
13. Have you ever observed any bleeding after brushing or flossing, or due to other conditions in your mouth?	☐ Yes ☐ No	☐ Yes ☐ No
14. Have you ever had any teeth become loose on their own, without an injury?	☐ Yes ☐ No	☐ Yes ☐ No
15. How many of your permanent teeth have been removed because of tooth decay or gum disease?	-----------------	☐ ≥ 1 tooth ☐ < 1 tooth
